# COVID-19 rapid diagnostic test could contain transmission in low- and middle-income countries

**DOI:** 10.4102/ajlm.v9i1.1255

**Published:** 2020-09-30

**Authors:** Adesola Olalekan, Bamidele Iwalokun, Oluwabukola M. Akinloye, Olayiwola Popoola, Titilola A. Samuel, Oluyemi Akinloye

**Affiliations:** 1 Department of Medical Laboratory Science, University of Lagos, Idiaraba, Lagos, Nigeria; 2 Centre for Genomics of Non-Communicable Diseases and Personalized Healthcare (CGNPH), University of Lagos, Akoka, Lagos, Nigeria; 3 Department of Molecular Biology and Biotechnology, Nigerian Institute of Medical Research, Yaba, Lagos, Nigeria; 4 Department of Medical Laboratory Science, Oulton College, Moncton, New Brunswick, Canada; 5 Department of Biochemistry, University of Lagos, Idiaraba, Lagos, Nigeria

**Keywords:** Coronavirus disease, COVID-19, SARS-CoV-2, rapid diagnostic test, low- and middle-income countries

## Abstract

**Background:**

Coronavirus disease 2019 (COVID-19) caused by severe acute respiratory syndrome coronavirus-2 (SARS-CoV-2) has impacted heavily on global health. Although real-time polymerase chain reaction (RT-PCR) is the current diagnostic method, challenges for low- and middle-income countries (LMICs) necessitate cheaper, higher-throughput, reliable rapid diagnostic tests (RDTs).

**Objective:**

We reviewed the documented performance characteristics of available COVID-19 RDTs to understand their public health utility in the ongoing pandemic, especially in resource-scarce LMIC settings.

**Methods:**

Using a scoping review methodology framework, common literature databases and documentary reports were searched up to 22 April 2020, irrespective of geographical location. The search terms included ‘SARS-CoV-2 AND serological testing’ and ‘COVID-19 AND serological testing’.

**Results:**

A total of 18 RDTs produced in eight countries, namely China (6; 33.33%), the United States (4; 22.22%), Germany (2; 11.11%), Singapore (2; 11.11%), Canada, Kenya, Korea and Belgium (1 each; 5.56%), were evaluated. Reported sensitivity ranged from 18.4% to 100% (average = 84.7%), whereas specificity ranged from 90.6% to 100% (average = 95.6%). The testing time ranged from 2 min to 30 min. Of the 12 validated RDTs, the IgM/IgG duo kit with non-colloidal gold labelling system was reported to elicit the highest sensitivity (98% – 100%) and specificity (98% – 99% for IgG and 96% – 99% for IgM).

**Conclusion:**

We found reports of high sensitivity and specificity among the developed RDTs that could complement RT-PCR for the detection of SARS-CoV-2 antibodies, especially for screening in LMICs. However, it is necessary to validate these kits locally.

## Introduction

Coronavirus disease 2019 (COVID-19) is an emerging respiratory disease that was first reported to the World Health Organization (WHO) as a cluster of pneumonia of unknown origin from Wuhan, China, in December 2019.^1^ The unknown causative agent was found through deep sequencing to be severe acute respiratory syndrome coronavirus-2 (SARS-CoV-2) on 7 January 2020 and the disease COVID-19 was named on 11 February 2020. In response, WHO declared COVID-19 as a Public Health Emergency of International Concern on 30 January 2020 and a pandemic on 11 March 2020.^1^ As of 22 April 2020, an estimated 2 572 805 confirmed cases and 178 551 confirmed deaths from COVID-19 had been reported.^2^ The first 10 cases in Africa were reported in five countries (Nigeria, Algeria, Morocco, Egypt and Senegal).^3^ Although earlier cases of COVID-19 in many low- and middle-income countries (LMICs) were described as imported by travelers from China, Italy, the United Kingdom and Germany, community transmission has now become the major cause of new COVID-19 infections.^3,4^ Early, rapid, large-scale diagnosis and accurate diagnosis of COVID-19 is one of the key interventions for COVID-19 containment in both high-income and LMIC settings.^3^ The availability of the SARS-CoV-2 genome has led to the development and validation of various reverse transcriptase real-time polymerase chain reaction (RT-PCR) *in vitro* diagnostic test kits by different manufacturers for COVID-19 diagnosis.^5,6^ This diagnostic test is based on the detection of genes encoding the envelope (E), spike (S), nucleoplasid (N), RNA-dependent RNA polymerase and open reading frame 1a/b (e.g. orf1ab, orf1a, orf1b) polyproteins within the genomic RNA of SARS-CoV-2.^5,6,7^ Due to lack of culture facilities, the RT-PCR method is currently the reference standard method of confirming COVID-19 diagnosis in suspected cases globally. For epidemiological investigation, public health and clinical actions, RT-PCR has been shown to be very reliable at screening and confirming the diagnosis of COVID-19 using upper respiratory (e.g. nasopharyngeal swab, oropharyngeal swab, throat swab and nasal swab) and lower respiratory (e.g. sputum and bronchioalveolar lavage) samples.^7,8^ Real-time PCR has also been useful for monitoring viral RNA shedding dynamics during the acute phase of the disease and viral RNA decay and disappearance during the convalescence stage of the disease among survivors.^9,10^ However, in spite of its high analytical sensitivity its detection range is limited to 3.2 – < 10.0 RNA copies per reaction.^6,7,8^ The RT-PCR method has been reported from studies done inside and outside China to also be prone to giving false negative results under certain conditions, thereby missing some COVID-19 cases. These missed cases are therefore not isolated increasing community transmission.^8,9,10^ These conditions include insufficient or inappropriate sample for viral RNA isolation, poor sample transportation to the laboratory, poor storage of the isolated RNA samples, poor quality of the RT-PCR assay and poor timing for sample collection. The asymptomatic phase of SARS-CoV-2 infection – the first few days post infection onset and the convalescence phase ≥ 14 days post infection onset, especially in a missed infection, have been indicated as times when cases can be missed.^7,8,9,10^ Poor quality RT-PCR assay is characterised by an inconsistent cycle threshold value and/or lack of amplification signal for one or two targeted genes. These missed cases are therefore not isolated increasing for SAR-CoV-2 detection.^6,7,8^ Also, due to limited financial resources, the limited number of accredited molecular laboratories of biosafety level 2/3 and limited number of technical experts, the scaling up of RT-PCR for COVID-19 diagnosis is limited in LMICs.^7,8,9,10^

**FIGURE 1:**
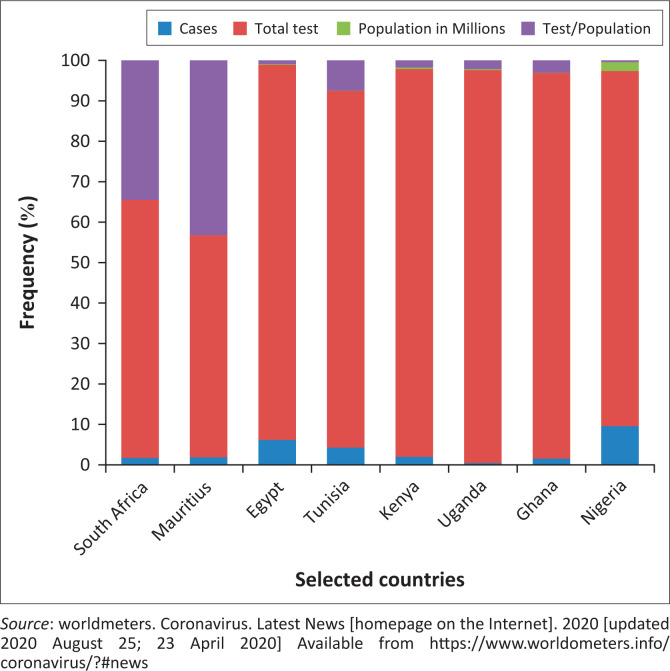
Distribution of severe acute respiratory syndrome coronavirus-2 burden and test per population in selected African countries.

Taken together, the above challenges of RT-PCR have necessitated the deployment of serological rapid diagnostic tests (RDTs) for COVID-19 diagnosis, which could identify asymptomatic and convalescent COVID-19 cases undiagnosed by RT-PCR. COVID-19 serological RDTs are antigen-antibody based tests that detects SARS-CoV-2 IgM and/or IgG in human blood samples or SARS-CoV-2 viral antigen from respiratory samples within 15 min.^8,11,12^ Unlike the RT-PCR protocols, serological tests require less expensive equipment, no technical expertise or electricity to run and very minimal biosafety requirements. Also, unlike RDTs that use small amounts of biological sample (10 uL – 20 uL) and have an average run time of 15 minutes, the RT-PCR protocols use large amounts of samples (150 ul – 200 ul) and have an average run time of about 90 minutes.^6,7,8^ These advantages of serological RDTs have attracted serious attention for their use in large-scale COVID-19 serological RDTs are antigen-antibody based tests that detects SARS-CoV-2 IgM and/or IgG in human blood samples or SARS-CoV-2 viral antigen from respiratory samples within 15 min testing especially at the peripheral level of the health system and outside hospital settings in LMICs. Data from *worldmeters* show that African countries compared to other countries conducted fewer tests per population (Figure 1). This lower testing power means relatively fewer cases can be detected. Thus, the rollout of various RDT kits by different manufacturers could be a favourable development particularly for LMICs as RDTs can be easily scaled up for rapid COVID-19 diagnosis.^11,12^ Besides, RDTs can provide additional sero-epidemiological data that will be used to determine the magnitude of COVID-19 spread within a population. RDTs achieve this by identifying active and previous symptomatic or asymptomatic cases; these data are then used to calculate case-fatality rate and determine the anti-SARS-CoV-2 immunity level of a community.^11,12^ However, to harness the various epidemiological and clinical usefulness of currently available COVID-19 serological RDTs, it is important to determine and/or validate their performance levels. In the present scoping review, the following research questions will be answered: (1) what are the currently available serological RDTs for testing, (2) to what extent have these serological RDTs been validated by their manufacturers and (3) what is the level of performance characteristics of these serological RDTs? Presently, the level of accuracy of many serological RDTs available for use in LMICs remains unclear, coupled with insufficient information about their strengths and limitations. This review will provide insight into the performance characteristics of these kits and enable evidenced-based decisions for their possible use in large-scale COVID-19 testing and containment strategies in LMICs.

## Methodology and data analysis

A scoping review was conducted using a methodology framework by Arksey^13^ with modification as described by Adhikari et al.^14^ This includes: (1) identifying a clear research objective and search strategies, (2) identifying relevant research articles, (3) selecting research articles, (4) extracting and charting of data, and (5) summarising, discussing, analysing and reporting the results. The online databases searched included Google Scholar, medRxiv, bioRxiv and PubMed, as well as documentary reports and white paper publications from relevant online websites including WHO, the United States Centers for Disease Control and Prevention (CDC) and the Nigeria Centre for Disease Control (NCDC) for information on new RDTs for COVID-19 published up to 22 April 2020. The search terms used include ‘SARS-CoV-2 AND testing’, ‘COVID-19 AND rapid test’ and ‘COVID-19 AND diagnostic kits’. Diagnostic kits published for the confirmation of other coronaviruses, such as the coronavirus associated with the 2003 SARS outbreak in Asia and Middle East respiratory syndrome-coronavirus, were excluded. All the members of the review teams were involved in paper search and selection and a consensus was reached through peer review. Duplicated publications and those with insufficient information were removed. The extracted data included the name of the diagnostic kit, manufacturer, test performance based on sensitivity, specificity, predictive positive and negative values, test principles and special characteristics and testing time. The data were entered into Excel (Microsoft, Redmond, Washington, United States) and exported to Statistical Product and Service Solution version 23 (IBM SPSS Inc., Chicago, Illinois, United States) for cleaning and analysis.

## Review findings

**FIGURE 2:**
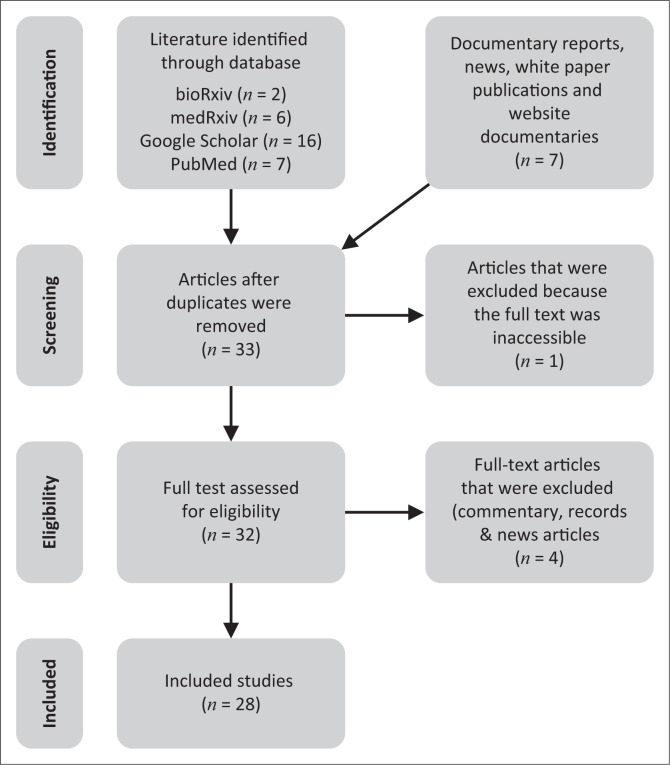
PRISMA flow diagram showing the scoping review process.

**FIGURE 3:**
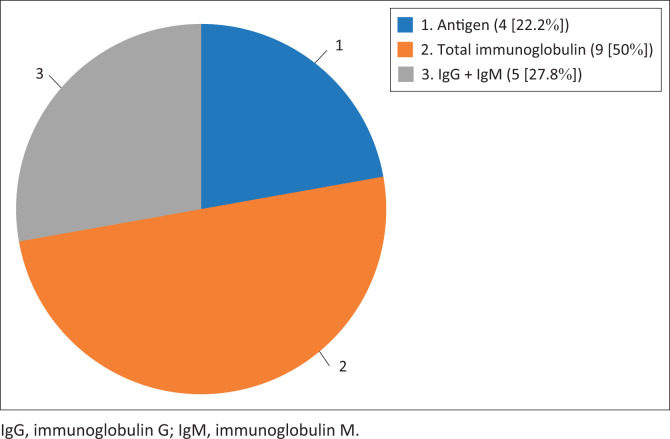
Distribution of the serological rapid diagnostic tests by testing principle.

**FIGURE 4:**
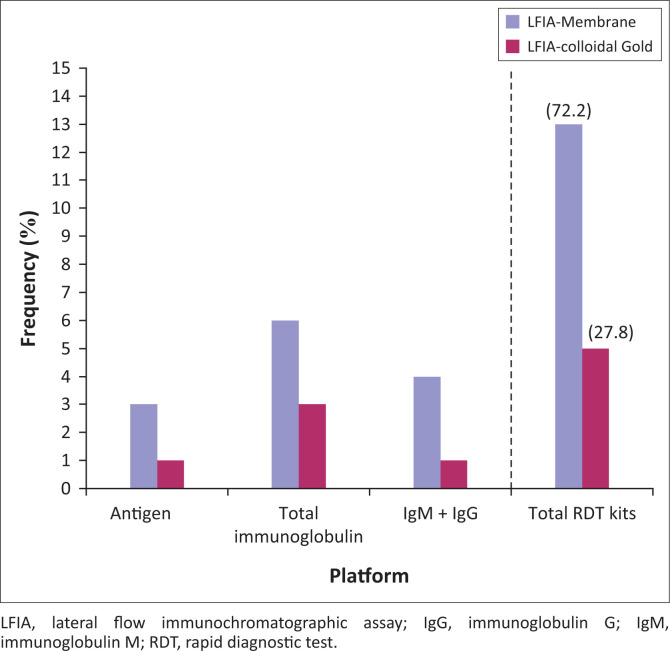
Distribution of the serological rapid diagnostic tests by testing platform.

Overall, 28 publications on coronavirus-based diagnostic kits that matched the goal of this publication were included in this study (Figure 2). Articles were excluded based on duplication and lack of information on detection principle, type of kit, performance characteristics and manufacturers’ details. All eligible publications on COVID-19 diagnostic kits by country and performance as of 22 April 2020 were summarised in numbers and percentages using descriptive analysis. On the whole, a total of 18 serological RDT kits were included for analysis. Of these, four were antigen RDTs (22.2%), nine were total immunoglobulin RDTs (50%) and five were IgM/IgG serological RDTs (27.8%) (Figure 3). These kits were produced in eight countries, namely China (6; 33.33%), the United States (4; 22.22%), Germany (2; 11.11%), Singapore (2; 11.11%) and Kenya, Canada, Korea and Belgium (1 each; 6.56%) (Table 1). Fourteen of the RDT kits are antibody-detection kits for use with blood, plasma or serum (77.8%), and four were antigen-detection kits for use with swab, sputum or blood (22.2%). The majority of these kits (13; 72.22%) use lateral flow membrane technology, whereas the remaining five (27.78%) use colloidal gold (Figure 4).

**TABLE 1:** Performance characteristics of newly developed severe acute respiratory syndrome coronavirus rapid diagnostic kits analysed in this review, 22 April 2020.

Serial no.	Name of kit	Manufacturer	Performance characteristics	Methods and specimen	Recorded comparison to real-time polymerase chain reaction	Time	References
**Antigen detection based rapid diagnostic testing kits**							
1.	Covid-19 Ag Resp-Strip	Coris BioConcept Belgium	Spe: 100.0% Sen: 96.0% PPV: 100.0% NPV: 96.2%	LFIA membrane based – Nasopharyngeal	-	15 min	^34^
2.	COVID 19 Rapid test kit	KEMRI, Research Institute, Kenya	Not available	LFIA membrane based – Swab	Shorter test time	15 min	^35^
3.	Camtech Novel Coronavirus (COVID-19) Antigen Test	Camtech Diagnostics Pte Ltd, Singapore	Not available	Lateral Flow Colloidal Gold Immunochromatographic – blood/sputum	Include the use of reader (POCT device)	30 min	^36^
4.	Standard TM Q COVID-19 Ag Test	SD Biosensor, Inc, Republic of Korea	Spe: 97.7% Sen: 84.4%	LFIA membrane based – Nasopharyngeal	Cross reaction with SARS coronavirus and some chemicals	30 min	^37^
**Antibody based (total immunoglobulin) rapid diagnostic testing kits**							
5.	Bodysphere IgM/IgG Rapid test	Bodysphere Los Angeles, US	Spe: 91.0% Sen: 99.0%	LFIA membrane based (IgG & IgM) – Blood, Plasma and Serum	Shorter test time	2 min	^38^
6.	COVID-19 total antibody test Assay	Ortho Clinical Diagnostics, Raritan, New Jersey, US	Not available	LFIA membrane based – blood	For use in immunodiagnostic & integrated systems	Not stated	^39^
7.	SARS-CoV-2 rapid IgG-IgM antibody test kit	Jiangsu Medomics Medical Technologies, China	Spe: 90.6%, Sen: 88.7%	LFIA membrane based (IgM & IgM) – blood	Ease of use and POCT with no additional device	15 min	^40^
8.	SARS-CoV-2 rapid IgG-IgM combined antibody test kit	Euroimmun Medical Laboratory Diagnostics & Epitope Diagnostics, Germany	Non available	LFIA membrane based (IgM & IgG) – blood	Not stated	Not stated	^41^
9.	SARS-CoV-2 rapid IgG-IgM antibody test kit	Cold Spring Habour Laboratory, YHLO Biotech, Shenzhen, China	Spe: 90.6% Sen: 88.6 %	LFIA for combined immunoglobulin (IgM & IgG) – blood	Shorter test time	15 min	^42^
10.	One Step Novel Coronavirus (COVID-19) IgM/IgG	Artron Laboratories Inc, Canada	Spe: 97.7% Sen: 93.4%	LFIA membrane based (IgM & IgG) – blood	One step simple and easy to use cassette devises	Not stated	^43^
11.	VivaDiag COVID-19 IgM-IgG VivaChek	VivaChek Biotech (Hangzhou) Co., Ltd, China	Spe: 91.7% Sen: 18. 4%, NPV: 26.3% PPV: 87.5%	LFIA Colloidal Gold based IgM & IgG – serum or whole blood	Poorer Sen & NPV despite comparable Spe & PPV	15 min	^20^
12.	Colloidal Gold Immunochromatographic Assay combined (GICA)	Zhu Hai Liv Zon Diagnostics Inc, China	Spe: 100.0% Sen: 82.4%	Lateral Flow Colloidal Gold Immunochromatographic based – blood	Shorter test time	15 min	^44^
13.	ThermoGenesis’ Rapid COVID-19 Serological Test Kit	ThermoGenesis Holdings, Inc. Wendy Samford, US	Spe: 100.0% Sen: 99.1%	Lateral Flow Colloidal Gold Immunochromatographic –blood/ serum/ plasma	PCR-positive and negative patient blood samples indicate high reliability	5 min	^45^
**Antibody based (IgG) or/and (IgM) separated rapid diagnostic testing kit**							
14.	All TestR 2019-nCOV IgG/IgM Rapid Test Cassette	Hangzhou AllTest Biotech Co. Ltd, China	IgG Spe: 98.0% Sen: 100.0% IgM Spe: 96.0% Sen: 85.0%	LFIA membrane based (IgG/IgM) antibodies – serum, plasma and whole blood	Hematocrit level needs to be within 25–65%, cross reactivity with other virus & interferences with other substances	10 min	^46^
15.	BioMedomics COVID-19 test kit	Becton Dickinson New Jersey, US	Not available (Ongoing)	LFIA membrane based (IgM/IgG) – blood	Shorter test time	15 min	^47^
16.	Camtech COVID-19 IgM/IgG Cassette	Camtech Diagnostics Pte Ltd, Singapore	Reported as fast and simple but details not available	LFIA membrane based (IgM/IgG) – blood	Humidity affects the stability of the kit	10 min	^36^
17.	NADALR COVID-19 IgG/IgM Test Cassette	Nal von minden GmbH, Germany	IgG Spec: 99.0% Sen: 98.0% IgM Spec: 99.0% Sen: 94.0%	LFIA membrane based (IgG/IgM) – whole blood, plasma or serum	Affected by temperature and cross-reaction with other viruses and interference with several chemicals.	10 min	^48^
18.	Colloidal Gold Immunochromatographic Assay (GICA)	Zhu Hai Liv Zon Diagnostics Inc, China	IgG Spe: 100.0% Sen: 81.3% IgM: Spec: 100.0% Sen: 57.1%	Lateral flow Colloidal Gold Immunochromatographic based antigen – antibody in blood	Shorter test time	15 min	^44^

Spe, specificity; Sen, sensitivity; NPV, negative predictive value; PPV, positive predictive value; LFIA, lateral flow immunochromatographic assay; SARS, severe acute respiratory syndrome; CoV, coronavirus; POCT, point of care testing; IgG, immunoglobulin G; IgM, immunoglobulin M; US, United States.

In general, the sensitivity of the test kits irrespective of sample specification ranged from 18.4% to 100% and their specificity ranged from 90.6% to 100%. The pooled analysis revealed an average (range) sensitivity of 81.6% (72.9% – 88%) and specificity of 94.4% (88.2% – 97.5%). The sensitivity and specificity of lateral flow immunoassay membrane type RDT kits were in the range (average) of 84.4% – 100% (92.7%) and 90.6% – 100% (96%), respectively, and that of lateral flow immunoassay colloidal gold type were 18.4 – 99.1% (67.7%) and 91.7% – 100% (98.3%), respectively (Figure 4). Three of these kits, namely Bodysphere Rapid Test (Los Angeles, California, United States), Thermogenesis Rapid COVID-19 Test kit (Rancho Cordova, California, United States) and NADAL® COVID-19 Test kit (Regensburg, Germany), had a sensitivity of 99% – 100%. These three kits also had a specificity range of 91% – 100%. Asides their better sensitivity and specificity compared to other RDTs, these kits are for use with blood samples only, detect both IgG and IgM, and have shorter testing time of 2 – 10 min. The testing time for all the identified kits ranged from 2 to 30 min with an average testing time of 13.5 min (95% confidence interval = 10.8 min – 16.1 min). Only two of the kits provided information on positive predicted value and negative predictive value (range = 87.5% – 100.0% to 26.2% – 96.2%).

**FIGURE 5:**
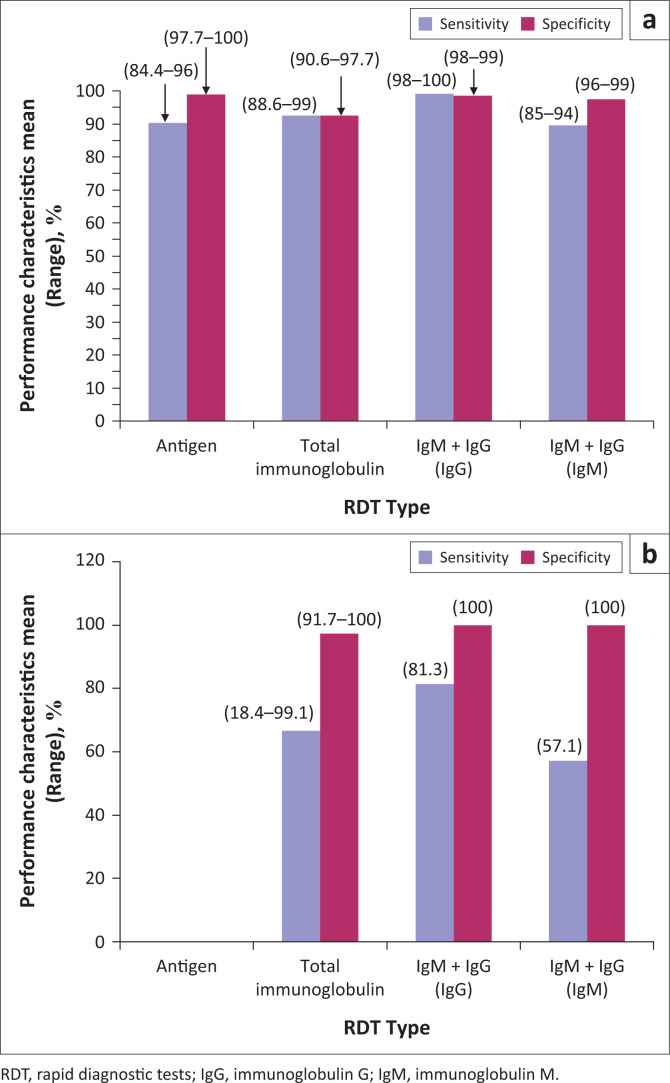
Performance characteristics of the different serological rapid diagnostic tests by testing platform; LFIA membrane (a) and colloidal gold devices (b).

Out of the 18 RDTs identified, 6 (33%) were not subjected to performance validation by the manufacturers of the kits. Two of four antigen detection kits, seven of nine total immunoglobulin and three of five IgM + IgG serological kits were validated for sensitivity and specificity using RT-PCR assay as the reference method (Table 1).^15^ On the whole, eight of the 13 lateral flow immunoassay membrane type and four of the five lateral flow immunoassay colloidal gold type kits were validated. Of the 12 serological RDTs validated by RT-PCR, the IgM/IgG duo kit with non-colloidal gold labelling system was found to elicit the highest and acceptable sensitivity (98% – 100%) and specificity (98% – 99%) values for IgG and specificity of 96% – 99% for IgM compared to other RDT types and the counterpart colloidal gold system-based IgM/IgG duo kit (Figure 5).

## Implications and recommendations

The need to expand diagnostic testing in order to cope with the current spread of COVID-19 infection in many settings in LMICs where resources for RT-PCR are limited and difficult to sustain has made RDT kits for SARS-CoV-2 an important tool in the global fight against the COVID-19 pandemic. For patients with suspected infection, RT-PCR is used to detect SARS-CoV-2 in sputum, throat and nasopharyngeal swab, and secretions of the lower respiratory tract samples such as bronchoalveolar lavage and bronchial washings.^16,17,18^ However, limited facilities and human resources for molecular testing using RT-PCR tends to slow down testing for COVID-19 in resource-limited countries. It has been argued that RDTs do not have sufficient evidence to support their use in the COVID-19 pandemic and hence should be used only in a research setting.^19^ Cassaniti et al. have earlier reported low sensitivity and specificity of serological assay which led to misdiagnosis of COVID-19 in the vast majority of the patients in their study population.^20^ The WHO has emphasised that tests with inadequate quality may miss patients with active infection or falsely categorise patients as having the disease, further hampering disease control efforts, hence the need for questioning the performance of SARS-CoV-2 RDT kits.^19,21^ Most manufacturers of the RDTs have performance characteristics of the kits validated using the RT-PCR technique as the reference method. However, several publications have reported the possibility of false-negative results using RT-PCR.^22^ Thus, the sensitivity and specificity data of reviewed kits should be understood in light of this bias.

The declaration of COVID-19 as a global pandemic and the huge concern of its transmission in LMICs where HIV, tuberculosis and malaria are currently endemic have necessitated the need to scale up diagnostic testing to mitigate further spread and the rising number of COVID-19 deaths outside China.^23,24^ In many settings in LMICs, such as small communities, riverine areas, health posts and primary health centres, resources for RT-PCR are absent.^23,24^ This has made the development of serological RDTs for the detection of specific SARS-CoV-2 antigens, anti-SARS-CoV-2 IgM and anti-SARS-CoV-2 IgG an attractive and very important tool in the global fight against the COVID-19 pandemic in LMICs. Findings from the 18 serological RDT kits analysed in this review imply that three different types of serological RDTs, antigen, total immunoglobulin, and combined IgM and IgG-based RDT with the ability to provide results between 2 min and 30 min are currently available for potential large-scale testing in LMICs using five types of biological samples (nasopharyngeal swab, throat swab, whole blood, plasma and serum). Due to challenges associated with more sensitive biological samples such as bronchoalveolar lavage and sputum, both nasopharyngeal and throat swabs are used for COVID-19 testing by RT-PCR in many settings.^7,8,9^ Also, whole blood, plasma or serum is often used as biological sample for RT-PCR for monitoring viremia to predict COVID-19 severity during the acute stage of infection and viral clearance during the convalescent stage.^7,8^ The latter is currently used to inform hospital discharge decisions in many countries; use of different samples for diagnosis and viral clearance determination can negatively impact on discharge decision-making.^8,9,10,12^ A potential way of circumventing discharge decision errors is to employ a diagnostic tool that uses the same type of sample for both diagnosis and viraemia monitoring such as the SARS-CoV-2 antigen and specific anti-SARS-CoV-2 IgM/IgG duo detection kit identified in this review. This can be integrated into the local COVID-19 management guidelines in LMICs. This guideline is currently being used in Malaysia and Europe.^25,26^ The primary weakness of RT-PCR for COVID-19 diagnosis lies in its inability to detect infection using nasopharyngeal samples collected outside the viral RNA shedding period. The shedding period is characterised by presence of low viral RNA, such as seen in asymptomatic, pre-symptom days (~2 days prior to symptom onset) and post-infection days (~14 post infection onset).^26,27^ Also, the RT-PCR, may also miss infections due to poor sample collection and preparation as well as poor storage of isolated RNA. These weaknesses can be addressed by serological RDTs, which detect the more stable viral immunogenic proteins such as the S and N proteins, which persist more than RNA or anti-SARS-CoV-2 IgM and IgG which have been reported to peak between 2 and 3 weeks and 17 days post infection onset.^26,27^ Guo et al.^28^ reported an improvement of COVID-19 identification by RT-PCR from 51.9% to 98.6% with the integration of an IgM-based immunoassay. However, the results of sensitivity (18.4% – 100%) and specificity (90.6% – 100.0%) reported for 12 of the 18 reviewed serological RDT kits by their manufacturers imply that the currently available COVID-19 RDTs are not equally accurate and only a few of them pass the sensitivity and specificity benchmark of 95%. Zainol et al.^29^ recently reported a sensitivity range of 72.7% – 100.0% and specificity range of 98.7% – 100.0% for IgM/IgG duo-based serological RDT kits for COVID-19 in their review in which nine serological kits were analysed. The authors also reported a sensitivity range of 86.4% – 90.6% and a specificity of 99% for total immunoglobulin-based RDTs. In Brazil, Castro et al.^30^ reported a mean (range) anti-SARS-CoV-2 IgM sensitivity of 82% (76% – 87%) and specificity of 97% (96% – 98%) and anti-SARS-CoV-2 IgG sensitivity of 97% (90% – 99%) and specificity of 98% (97% – 99%). Although in this review, only 5 of the 18 serological RDT kits offered combined IgM and IgG detection, we also found a better performance characteristic for this type of RDT kit compared to the antigen and total immunoglobulin kits using non-colloidal gold labelling system with acceptable sensitivity (98% – 100%) and a specificity (98% – 99%) values for IgG and specificity of 96% – 99% for IgM, suggesting the ability of these kits to detect past infections, confirm true negative results and rule out false positive COVID-19 testing results by RT-PCR. However, the performance of these kits to confirm recent infections seems to be below the benchmark of 95%, since they had a sensitivity range of 85% – 94%, which was even lower for colloidal gold labelling systems at 57.1%. Meanwhile, the improvement offered by the antigen-based RDT kits in this review can be said to be none or marginal at 84.4% – 96%. Another implication of these findings is that more than one serological RDT kit may be needed for a SARS-CoV-2 detection algorithm to improve confirmation and diagnosis of COVID-19 by RT-PCR, if deployed in LMICs. It is also important to note that 6 of the 18 reviewed serological RDT kits lacked reports on sensitivity and specificity, thus the accuracy in diagnosising COVID-19 is unknown as at the time of this review. This finding further reiterates the difficulty associated with SARS-CoV-2 serological RDT kit validation by manufacturers, since RT-PCR the reference method targets viral RNA instead of specific SARS-CoV-2 antibodies or antigens. A similar opinion has been shared by Castrol et al.,^30^ given the well-documented differences in the kinetics of the viral RNA (even between samples) and anti-SARS-CoV-2 antibodies in infected individuals.

As of 01 April 2020, the death toll for COVID-19 was over a million globally and the need for accurate intervention to stop transmission and re-infection of COVID-19 is now extremely necessary. The WHO advises countries to improve the rate of testing to identify an infected individual for appropriate isolation and treatment. The availability of efficient and rapid diagnostics for COVID-19 has been indicated as one of the mitigation strategies to control the pandemic. Rapid diagnostic tests are cheaper and more readily available; thus, they might be more useful stopping transmission by rapidly identifying positive and previous cases particularly in LMICs. These data will in turn be useful for both disease diagnosis and surveillance. The RDT will either detect the presence of viral proteins (antigens) expressed by the COVID-19 virus or the presence of antibodies in the blood of COVID-19-infected people.^31,32^ The performance of the kits has been shown to depend on several factors such as the onset of illness, the viral load in the specimen, the integrity of the specimen collected from suspected cases, processing, age, nutritional status, the severity of the disease, and certain medications or underlying disease condition, especially immune suppression diseases and the precise formulation of the reagents in the test kits.^19^

The LMICs reported the lowest rate of testing per population with corresponding lower numbers of cases compared with developed countries. This may be an indication of limited testing resources and facilities due to the challenges associated with RT-PCR. Therefore, there may be several cases in this population that are not detected with antecedent clinical implications. The use of RDTs will not only help to detect currently infected or previously exposed individuals who have developed immunity as well as identify asymptomatic carriers. These will inform decisions for public health measures, for example, cases among a more IgM-positive population may be an indication of a subclinical outbreak. The economic impact of movement restrictions and lockdowns in many of these countries is not well managed, adding unimaginable suffering in an already impoverished population. The use of RDTs for the screening of COVID-19 may help to determine individuals who are at lower risk and may be permitted to go back to work. When coupled with clinical symptoms and molecular testing, RDTs may serve as a first-line tool for diagnosis and help to better understand the spread of diseases.

Although the COVID-19 test kit market is in its infancy, the global COVID-19 outbreak and up-surging cases are driving the demand for RDTs, hence researchers throughout the world are striving to develop RDTs to track infected people. To date, very few countries have succeeded in developing SARS-CoV-2 testing kits, while some are still working on improving the performance of their products. With the dedicated global efforts on preventing the spread of COVID-19 and flattening the curve, significant improvement must have occurred in improving the performance of COVID-19 test kits. Increasing accessibility to testing among other interventions has improved the containment and transmission of the infection. While algorithms have been developed to limit testing to individuals that fulfil certain criteria, such as contact with the infected patients, clinical symptoms of COVID-19, travelling history to epidemic countries, etc., testing the entire population has been recommended.^33^ Resource limitations means most LMICs can not cope with the up-surge of infection and transmission. While testing per population is high in developed countries with over 3 million tested in the United States, testing per population is still very low in developing countries with less than 10 000 tested in Nigeria as of 22 April 2020. Therefore, the addition of validated serological-based RDT even with lower performance characteristics compared to RT-PCR may serve as complementary tools to increase the rate of testing per population, especially in LMICs where community transmission is now on the rise. Therefore, the use of RDTs operated as lateral flow immunochromatographic assays to detect both IgM and IgG on separate test lines using whole blood, plasma or serum samples is desirable for LMICs. Wang et al. reported that the combination of RT-PCR testing and clinical features for diagnosis of COVID-19 facilitated the management of the SARS-CoV-2 outbreak in China,^22^ and COVID-19 mass testing facilities have been strongly advocated to end the epidemic rapidly.^34^ The use of RDT will not only allow mass testing facilities in LMICs but coupled with clinical features in symptomatic patients and molecular testing (RT-PCR) in asymptomatic populations may help to contain transmission in LMICs.

### Conclusion

Considering the peculiarity of LMICs, especially their economic situation, the standard RT-PCR may not be able to cope with the testing needs of these countries because of limited infrastructure and human resources. Generally, it is agreed that rapid testing techniques are useful for screening for early detection of symptomatic cases, which is crucial for averting community or hospital transmission and strengthening contact tracing and active surveillance. This review revealed considerable good performance of the RDT with manufacturer sensitivity and specificity using varieties of samples including blood samples. Hence, the use of RDT kits in LMICs may increase access to testing and better triaging of COVID-19 patients. We, however, identified that most of the proposed rapid kits have not been optimised and validated. It is important that the kits undergo further validation with samples from countries of proposed use in reference to RT-PCR before use.
